# Salvage for cervical recurrences of head and neck cancer with dissection and interstitial high dose rate brachytherapy

**DOI:** 10.1186/1748-717X-1-27

**Published:** 2006-08-08

**Authors:** Antonio Cassio Assis Pellizzon, João Victor Salvajoli, Luiz Paulo Kowalski, Andre Lopes Carvalho

**Affiliations:** 1 Radiation Oncology Department, Centro de Tratamento e Pesquisa, Hospital ACCamargo, São Paulo, Brazil; 2 Head and Neck Surgery Department, Centro de Tratamento e Pesquisa, Hospital ACCamargo, São Paulo, Brazil

## Abstract

Salvage therapy in head and neck cancer (HNC) is a controversy issue and the literature is scarce regarding the use of interstitial high-dose rate brachytherapy (I-HDR) in HNC. We evaluated the long-term results of a treatment policy combining salvage surgery and I-HDR for cervical recurrences of HNC. Charts of 21 patients treated from 1994 to 2004 were reviewed. The crude local control rate for all patients was 52.4%. The 5- and 8-years overall (OS) and local relapse-free survival (LRFS) rates were 50%, 42.9%, 42.5% and 28.6%, respectively. The only predictive factor associated to LFRS and OS was negative margin status (p = 0.0007 and p = 0.0002). We conclude that complete surgery is mandatory for long term control and the doses given by brachytherapy are not high enough to compensate for microscopic residual disease after surgery.

## Background

The best approach for advanced head and neck cancer (HNC) is the combined modality using surgery, adjuvant radiotherapy with or without chemotherapy. For patients who refuse surgery, the superiority of radiation (RT) concurrent with chemotherapy (CHT) in local and regional tumor control has been established in several randomized studies and meta-analyses [1,2], but even for multimodality treated patients the recurrence rates above the clavicles occur in up to 20% of patients [3,4].

Salvage therapy in HNC is still a controversy issue and the best combination approach is still to be defined. It seems that maximum debulking surgery combined to a primary or a second new course of RT can lead to a better local control (LC). Conversely, a new course of external beam radiotherapy (EBRT) for recurrent disease is always a problem and of limited feasibility because of the difficulty to spare adjacent normal tissues, resulting in undesirable late effects on the salivary glands, mandible, and muscles of mastication. In these cases the use of intra-operative interstitial implantation is an option, as it is ideally suited to deliver a high dose to a limited volume, thus minimizing sequelae and improving LC [5]. I-HDR can also increase total biological effective dose administered when compared to a second course of EBRT, the overall time is decreased and it is also a very conformal way of treatment, allowing protection of normal surrounding structures [6].

In 1996 we started an institutional treatment policy of post-operative I-HDR for recurrent cervical carcinomas, with tumor control on the site of the primary, to take advantage of shortening the overall treatment time and conformability of the procedure.

## Patients and methods

All patients admitted for treatment at the Radiation Oncology and Head and Neck Surgery Departments, Hospital do Cancer A.C. Camargo, São Paulo, Brazil, from October 1994 to June 2004, were retrospectively selected. The criteria for including patients in the study were: recurrent cervical cancer with local control of the primary site, biopsy proven squamous cell carcinoma (SCC), Karnofsky performance 60 and above, possibility of surgical resection no evidence of distant metastasis. A total of 21 patients were selected and the clinical stage defined based on preoperative clinical and radiological examinations, using the TNM classification of the AJCC [7]. Clinical characteristics of patients at presentation are summarized in Table 1.

**Table 1:** Characteristics of patients with cervical recurrences and local control of the tumor primary site

Variables	n. pacients	%	median
*Age*			53.5
*< 65 years*	15	71.4	
≥ *65 years*	6	28.6	
*Gender*			
*Male*	16	76.2	
*Female*	5	23.8	
*Primary Tumor Site*			
*Cervical*	1	4.8	
*Face *(*skin SCC*)	4	19.0	
*Pharynx*	10	47.6	
*Oral cavity*	6	28.6	
*Lymph node mobility*			
*Mobile*	12	57.1	
*Reduced*	6	28.6	
*Fixed*	3	14.3	
*Previous Radiation*			
*Yes*	15	71.4	
*No*	6	28.6	

*Previous Chemotherapy*			
*Yes*	01	4.8	
*No*	20	95.2	

*Total*	21	100	

We observed that the initial evaluation for all patients consisted of a history and physical evaluation, including a careful locorregional exam, chest X Rays, and routine serum laboratory studies (complete blood count, biochemistry panel) and detailed information about the first course of EBRT. After a multidisciplinary team evaluation and the approval of a Review Board the procedure was performed.

All patients underwent to complete gross tumor resection. The details of treatment have been published elsewhere [8]. In summary, the treatment policy was as follows: all patients should have a pretreatment evaluation of the tumor target volume and normal tissue volumes at risk at Radiation Oncology Department. At the moment of the surgery, plastic catheters were inserted as parallel as possible, 10–15 mm apart one of each in the tumor bed with 15 to 20 mm margin. In a first moment, metallic markers were also inserted to help on defining the target volume at the planning system. Grafts were used, when indicated, to cover the tumor bed and to spare vascular structures and nerves or to overlay the catheters. Most patients had single plan implants, but whenever necessary biplane implants were performed.

**Figure 1:**
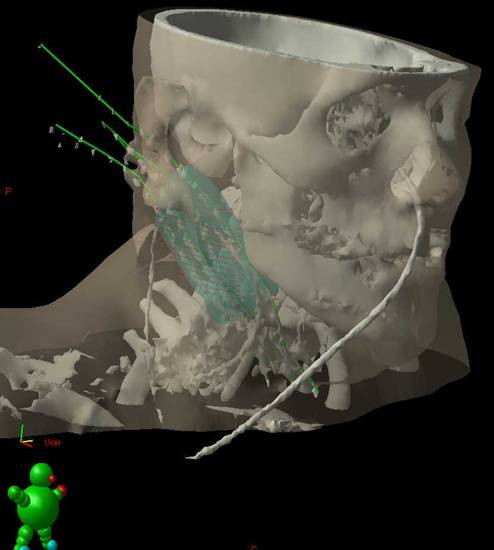
Three-dimensional planning CT image reconstruction.

For I-HDR planning and reconstruction, radiographs or CT scans were used in order to calculate exactly the dose distribution to the target volume and adjacent healthy tissues (Figure 1). The micro-Selectron HDR, Nucletron, or Gammamed-Varian equipment, with iridium-192 sources were used for treatment delivery (Nucletron B.V., Netherlands and Varian, Palo Alto, US). Plans were optimized using standard geometric optimization, and prescription dose was based on the Paris dosimetry system. Dose prescriptions were at the isodose that encompassed the tumor bed with a safety margin of 5 mm (median: 87%, range: 75–95%). Hot spots should not exceed 135% of prescribed dose, in one fourth of total irradiated target volume. Doses at the skin were measured and should not exceed 60% of the prescribed dose. The doses to spinal cord were measured and limited by the presence of a previous EBRT course. No patient had a nominal total dose above 48 Gy to the spinal cord.

Fifteen of the patients who presented cervical recurrence had had a previous course of external beam radiation with doses ranging from 30 to 66 Gy (median 52 Gy). For the second course of EBRT photons of 4 MV or 6 MV were used. The EBRT course usually started immediately after the completion of the course of I-HDR.

### Statistical analysis

The actuarial survival rates were calculated by the Kaplan Meyer method. Chi-square tests were used to find differences in proportions and the Log-rank test was used to compare equality of survivor functions. The Cox proportional risk model was used for establishing a multi-factorial model considering the variables with independent prognostic value and the T-test was used to compare means. The follow up time was defined as the interval between the date of being admitted to the hospital and the date of the last objective follow up information or death.

Statistical significance was considered for p-values < 0.05.

## Results

The median age of patients was 53.5 (range 31–73) years and the follow up time ranged from 6 to 82 months (median – 36 months). The median interval between first treatment and salvage therapy was 32 months (range 14–86). The characteristics of patients are shown in table 1.

There was a predominance of male patients (76.2%) and the ratio male to female was 3.2:1. Fifteen patients (71.4%) had previous EBRT with doses ranging from 30 Gy to 66 Gy (median 52 Gy).

The total dose of I-HDR for all patients ranged from 12 Gy to 48 Gy (median 35), given in 3 to 16 fractions (median 8 fractions), in a median of 4 days (range 3 to 8). The median interval between the surgical intervention and start of I-HDR was 5 days (range 4–12). The median number of catheters inserted was 4 (range, 4–9) per patient. Two (9.5%) patients had double planar implants, and the remaining single plan implants.

The interval between the end of I-HDR and the start of the second course of EBRT ranged from 1 to 37 days (median 7 days). No patient had concurrent I-HDR and CHT, but one patient had concurrent platinum based CHT at time of EBRT course. The total treatment time, including the surgical procedure, ranged from 14 to 72 days (median 38 days).

All patients had combined EBRT and I-HDR. The median dose of EBRT was 45 Gy (range 30–66). For the previous irradiated patients the second course of EBRT ranged from 25 to 50 Gy (median 30 Gy), given in 1.8 to 2.0 Gy per fraction. Using the linear quadratic model we have calculated the Biological Effective Dose (BED) given to the tumor bed, assuming the α/β value of 10, for the EBRT, I-HDR, second EBRT course and the total BED for all courses of radiation, as shown in table 2.

**Table 2:** Doses and Biological Effective Doses for all patients

	Dose (Gy)				BED (Gy_10_)			
		
	EBRT	Second EBRT	I-HDR	Nominal Dose EBRT + I-HDR	EBRT	I-HDR	Second EBRT	Total
N	21	15	21	21	21	21	21	21
Median	45.0	30.0	21.0	65.0	53.1	31.0	35.4	111.2
SD	14.0	9.3	10.3	11.6	13.8	17.9	10.9	22.2
Min.	30.0	25.0	7.0	46	53.1	13.0	29,5	62.2
Max.	66,0	50.0	42.0	105.0	86.1	91.0	59.0	146.8

Legend – n – number; SD – standard deviation; Min – minimum; Max – maximum

For the 15 patients who had had a previous course of EBRT, the total I-HDR median dose was 24 Gy, inferior to the median dose of 40 Gy given to patients without a previous course of EBRT (p = 0.011), but with no influence in OS (p = 0.9436) or LRFS (p = 0.6579), respectively. The total value of BED above or inferior to 111 Gy_10 _also had no impact on OS (p = 0.264) or LRFS (p = 0.7686). Table 3.

**Table 3:** Local relapse-free rates

Variable	n. patients	Local control	%	P*
Age				
≤ 65 years	15	7	56.7	0.3330
> 65 years	6	4	73.3	
Primary site				
Face	4	2	50.0	0.3485
Pharynx	10	5	50.0	
Oral cavity	6	4	66.7	
Unknown	1	0	0	
Cumulative Dose				
≤ 70 Gy	7	5	71.5	0.8920
> 70 Gy	14	6	52.8	
I-HDR without EBRT				
Yes	8	5	63.5	0.9436
No	13	6	53.8	
I-HDR Total Dose				
≤ 18 Gy	5	3	60.0	0.4365
> 18 Gy	16	8	50.0	
Lymphnode mobility				
Mobile	12	6	50.0	0.8814
Reduced	6	4	66.7	
Fixed	3	1	33.3	
Lymph fixation				
Mobile	12	6	50.0	0.8966
Fixed	9	5	55.5	
Margin status				
Negative	13	11	84.6	0.0007
Positive	8	0	0	
Previous radiation				
Yes	15	8	53.3	0.6052
No	6	3	50.0	
BED (Gy_10_)				
≤ 111	10	4	40.0	0.7686
> 111	11	6	54.5	

Total	21	11	52.3	

**Figure 2:**
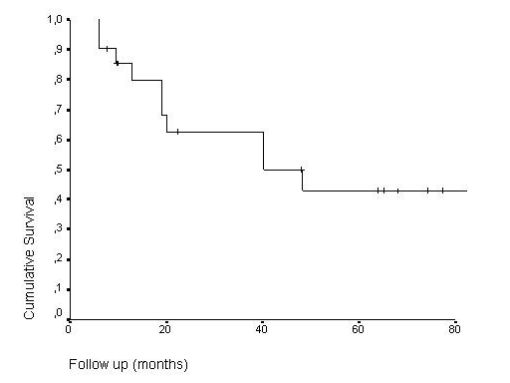
Kaplan Meier overall survival estimates.

**Figure 3:**
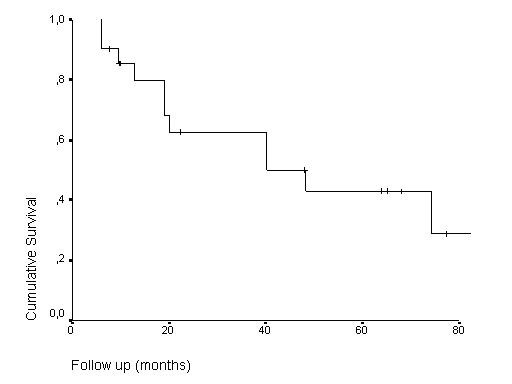
Kaplan Meier local relapse-free survival estimates.

The crude local control rate was 52.4% (11/21 patients). The 5- and 8-year overall (OS) and local relapse-free survival (LRFS) rates were 50%, 42.9%, 42.5% and 28.6%, respectively, as shown in Figures 2 and 3. There was no local failure within the irradiated area.

**Figure 4:**
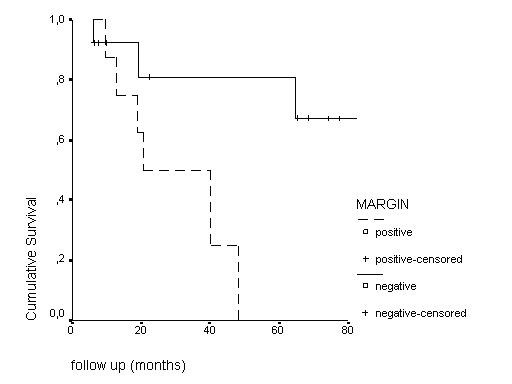
Kaplan Meier local relapse-free survival estimates by margin status.

Taking the data set of the 21 patients (Table 3), the only statistical significant prognostic factor for LRFS and OS at 5 and 8 years was margin status, p = 0.0007 and p = 0.0002, respectively. Figure 4.

Doses at skin were measured and where in the range of 32–60%. Four patients (19.4%) experienced acute and late adverse effects. Suture dehiscence occurred in 3 patients and one developed subcutaneous infection. Severe late side effects as local ulcer and extensive neck fibrosis were seem in 3 and 1 patient, respectively. There was no soft tissue necrosis in the series.

## Discussion

The presence of secondary neck node metastasis, as first presentation or recurrence, is an adverse event that impacts on survival. Salvage therapy in HNC is a controversy issue, but the best combination approach seems to be the maximum debulking surgery combined to irradiation (primarily or a second course). Conventional EBRT as a second course is generally limited by the tolerance of surrounding normal tissues. Three-dimensional conformal radiotherapy (3D-RT) or IMRT is time consuming, cost effective treatments, and there is a lack of facilities in developing countries [8].

The interstitial implantation is ideally suited to deliver a high dose limited to the volume of the primary tumor site, thus maximizing tumor control while minimizing complications. With conventional EBRT alone, it is difficult to spare adjacent normal tissues, resulting in undesirable late effects on the salivary glands, mandible, and muscles of mastication [9].

Regarding brachytherapy, a large experience has been accumulated with interstitial low dose rate (I-LDR). Grabenbauer et al published results of 318 patients treated between 1985 and 1997 by I-LDR as part of their primary (n = 236) or recurrent treatment (n = 82). A total of 175 patients (55%) received a combination of surgery, I-LDR (23–25 Gy) and EBRT (50–60 Gy), 60 patients (19%) surgery and I-LDR (45–55 Gy) alone. At 5 years they observed a better OS rate (p < 0.0001) for patients receiving primary therapy. Median LRFS rates were 74% and 57% at 5 years following primary and recurrent treatment (p = 0.01), respectively. Late treatment-related toxicity with soft tissue necrosis and/or osteonecrosis requiring mandibular resection was 7.5% [10].

Lapeyre et al. in a recent publication reviewed the indications of postoperative I-LDR performed with Iridium 192 for SCC of the oral cavity with positive or close margins. From 1979 to 1993, 82 patients were treated. Forty-six patients had combined EBRT (mean dose of 48 Gy) and I-LDR (mean dose of 24 Gy). Thirty-six patients had I-LDR alone with a mean dose of 60 Gy. OS and LC at 5 years were in the range of 22% to75% and 57% to 92%, respectively, Predictive factors for OS and LC were positive limphonodes (p = 0.009) and extra-capsular spread (p = 0.000001). The CS was only predictive for LC (p = 0.02) [11].

Many institutions replaced the I-LDR I-HDR, with preliminary results already published. I-LDR may improve the LC after surgery because it can increase total dose administered, decrease the overall time, and is very conformal, allowing protection of normal surrounding structures [6]. The literature is scarce regarding the use of I-HDR in treatment of cervical region, and most reports are focused on primary tumors of mobile tongue and oropharynx. A phase I/II clinical trial tried to attempt for the use of I-HDR as monotherapy for initial HN-SCC at the British Columbia Cancer Agency, Vancouver Clinic, Canada, was has been tested from 1989 to 1993. A total of 27 patients with T1-3 SCC from mobile tongue were treated with 7 fractions of 6.5 Gy, over a period of 3.5 days. The actuarial LRFS was 53% at 5 years, lower than comparable historical controls [12]. Takacsi-Nagy et al. published data regarding 21 patients with base of tongue tumors treated with association of surgery and I-HDR between 1993 and 1999. Seventeen patients with advanced stage cancer received I-HDR as a boost after 60–66 Gy EBRT and 4 patients with early stage (T1-2N0) were managed by brachytherapy as sole treatment. The mean doses for combined and sole I-HDR were 20 Gy (12–24 Gy) and 27 Gy (24–30 Gy), respectively. At a mean follow-up time of 32 months the local tumor control for the entire patient population was 62%. (13/21). The incidence of grade 2 or grade 3 mucositis were 48% and 52%, respectively [13].

We observed that 13 (61.9%) patients, in our analysis, had had a previous course of irradiation, in which the median total I-HDR dose was 25.8 Gy, inferior when compared to the median dose of 40.7 Gy given to patients without a previous course of EBRT (p = 0.011), but with no influence in OS (p = 0.9436). Four patients (19.4%) experienced acute and late adverse effects. Suture dehiscence was observed in 3 patients and one patient developed subcutaneous infection. Late side effects, graded as severe complication, as extensive neck fibrosis and local ulcer, happened in 1 and 3 patients, respectively, not related to a higher dose to the skin or graft. There was no soft tissue necrosis.

Leung et al. have published data regarding 19 patients with early stage oral tongue cancer as treated by I-HDR. The male-female ratio was 1:0.9, and the median age was 60 years, with a median dose given of 55 Gy in 10 fractions over 6 days. They observed that the 4-year local failure-free survival rate was 94.7% and that one patient treated with double planar implant had grade II necrosis of the soft tissue [16].

In our analysis the only predictive factor for LFRS and OS was the resection margin status, p = 0.0007 and p = 0.0002, respectively.

Some studies using pulsed-dose-rate (PDR) brachytherapy are also available. Strnad et al have already published the results of interstitial PDR with regard to local control and the incidence of side effects in patients with recurrences in a previously irradiated area. From 1997 to 2001, 43 patients received interstitial PDR brachytherapy. The dose per pulse ranged from 0.4–0.7 Gy. Sixteen of 43 (37%) patients also received cisplatin or carboplatin with 5-fluorouracil during the time of treatment and 13/43 (30%) received EBRT in a dose range from 20–67 Gy. The 2-year LFRS and OS were 68% and 49%, respectively. For patients treated with curative intention they were 80% and 66%, respectively. They observed no statistical difference in the probability of local recurrence in patients subgrouped by recurrent tumor vs. secondary primary tumors [15].

The Linear Quadratic Model is a toll that can make easy to compare different fraction schedules. Assuming an α/β of 10, the most common value used for SCC, we found a total BED in our analysis to be in the range of 66.2 to 148.6 Gy_10_. We observed that a higher BED was not a prognostic factor for LRFS (p = 0.7686) or OS (p = 0.264). Conversely, a publication by Levendag et al reporting the use of I-HDR and PDR as primary treatment for 38 patients presenting with tonsillar fossa and/or soft palate SCC treated between 1990 and 1994 using I-HDR or PDR. The median cumulative dose of I-HDR or PDR with or without additional EBRT was 66 Gy (range 55–73). They observed that 5 (13%) patients developed local failure with salvage surgery being possible in three of them and that neither BT scheme nor tumor site significantly influenced local control rates. Using the BED to compare results with a group of 72 patients with similar tumor characteristics and who underwent to EBRT alone they observed that interstitial radiation, T stage, N stage, overall treatment time and BED were significant prognostic factors for LRFS and OS at 3 years on univariate analysis. Using Cox proportional hazard analysis, only T stage and BED remained significant for LRFS (p < 0.001 and p = 0.008, respectively) [16].

The technological advances have made the use of I-HDR more precise and appealing. For developing countries its association with salvage surgery seems to be a reasonable option, especially because of the high number of centers that possesses the HDR after loading facilities, due the high incidence of cervix cancer, and the lack of IMRT or 3D-RT.

In conclusion, our results suggest that I-HDR can be recommended in selected patients presenting local recurrences or second primary carcinomas after previous EBRT, as it leads to a satisfactory local control rate with acceptable morbidity. For patients who underwent to partial resections dose should be increased to allow a better tumor control. When IMRT is not suitable, the combination of I-HDR and salvage surgery, when feasible, seems to the best salvage approach, but further studies are still awaited.
